# Analysis of selected risk factors for nodal metastases in head and neck cutaneous squamous cell carcinoma

**DOI:** 10.1007/s00405-014-3261-6

**Published:** 2014-09-13

**Authors:** Mateusz Szewczyk, Jakub Pazdrowski, Paweł Golusiński, Aleksandra Dańczak-Pazdrowska, Sławomir Marszałek, Wojciech Golusiński

**Affiliations:** 1 Department of Head and Neck Surgery, Greater Poland Cancer Centre, Poznan University of Medical Sciences, Garbary 15, 61-866 Poznan, Poland; 2 Department of Biology and Enviromental Studies, Poznan University of Medical Sciences, Dluga ½, 61-848 Poznan, Poland; 3 Department of Dermatology, Poznan University of Medical Sciences, Przybyszewskiego 49, Poznan, Poland; 4 Department of Rehabilitation in Internal Medicine, University School of Physical Education, Poznan, Poland

**Keywords:** cSCC, Head, Neck, Metastasis, Skin, Cancer, Squamous cell carcinoma, Regional, Recurrence

## Abstract

Cutaneous squamous cell carcinoma (cSCC) accounts for 20 % of all skin malignancies and 20 % of deaths. In contrast to mucosal SCC, treatment results are very good. However, regional metastases are present in 5–20 % of cases, and the prognosis for patients with metastases is 50 % lower. It has been reported that several risk factors are responsible for the head and neck lymph node regional metastasis, such as: poor cell differentiation, local recurrence, immunosuppression, and tumour dimension. Multivariate analysis of metastatic neck lesions in head and neck cSCC. Retrospective analysis of patients treated at our department for head and neck cSCC. The study includes 100 patients: 66 males (66 %) and 34 females (34 %), aged 26–98 years (mean age 74.6). The tumour was evaluated for: sex predilection, local recurrence, stage (according to 7th edition of American Joint Committee on Cancer TNM staging), differentiation, and site. Most patients (79 cases; 79 %) were treated for primary cSCC, while the other 21 patients presented local recurrence of cSCC. Neck metastases were diagnosed in five patients with primary cSCC and in three with recurrent cSCC. No distant metastasis was observed. The most common tumour location was the auricle (29 cases; 29 %). Neck dissection was performed most frequently in patients with lip tumours (17/22 cases; 77 %). Neck metastasis was diagnosed most often in patients with cSCC on the lip (2 patients) and buccal region (2 patients). The most common tumour location in males was the auricle (25/66 cases; 38 %) whereas in females the nasal and buccal regions were the most common locations, with 8 patients each (8/34 cases; 23 %). Neck dissection was performed in 20 of the 66 males (30 %) and in 12 of the 34 females (35 %). Neck metastasis was confirmed in 5 females (15 %) and 3 males (5 %). The most common histopathological tumour stage was G2 (57 cases; 57 %). Of the eight patients with confirmed neck metastasis, four had poorly-differentiated (histopathological stage G3). Thus, 4 of the 24 patients (17 %) with stage G3 tumours experienced metastasis. Our findings suggest that factors such as local recurrence, degree of cell differentiation, tumour dimension and/or location, can increase the risk of neck metastases. For this reason, in patients with such risk factors, neck dissection should be considered to evaluate for metastatic lesions.

## Introduction

Cutaneous squamous cell carcinoma (cSCC) accounts for 20 % of all skin malignancies, and 20 % of skin cancer deaths [1]. The incidence of cSCC has been rising in recent decades and, by some estimates, the incidence rate increases by 10 % each year [2]. Treatment of non-melanoma skin cancers is expensive: it is the fifth most expensive cancer-related treatment in the United States. The primary risk factor for developing cSCC is chronic sunlight exposure, mainly to UV-B rays.

Most cSCC lesions (approximately 90 %) are located in the head and neck region [3]. Treatment results are quite good, especially when compared to mucosal SCC. However, regional metastases are present from 5 to 20 % of cases, and the presence of such metastases reduces the prognosis by 50 %: 5-year overall survival in patients with confirmed regional metastases and ranges from 25 to 70 % [4].

Proven risk factors for head and neck regional lymph node metastasis include: poor cell differentiation, local recurrence, immunosuppression, or large primary tumour size. The synchronous presence of clinically or radiologically-confirmed neck metastases requires tumour resection, neck dissection, and, in selected cases, postoperative radiotherapy.

The aim of the present study was to evaluate the risk factors of developing neck metastases in a group of patients with head and neck cSCC.

## Materials and methods

Retrospective analysis of patients treated for head and neck cSCC at the Department of Head and Neck Surgery of the University of Medical Sciences in Poznan, Poland. The study included 100 patients: 66 males (66 %) and 34 females (34 %), aged 26–98 years (mean age 74.6). The following tumour-related characteristics were recorded: patient sex, local recurrence, stage (7th edition, AJCC on Cancer staging [5]), cell differentiation, and site. The following potential risk factors for neck metastases were also evaluated: >1 head and neck cSCC; head and neck basal cell carcinoma (BCC), history or coexistence of another malignancy; tobacco use; and immunosuppression.

## Results

Most patients (79 cases; 79 %) underwent treatment for primary cSCC, with the remaining 21 patients (21 %) were treated for local recurrence of cSCC without primary neck dissection (Table 1). Of the 32 patients considered candidates for simultaneous tumour resection and neck dissection, 25 had not undergone previous surgery for the tumour. In the other 7 patients with local recurrence, the recurrent tumour was resected with additional primary neck dissection. Neck metastases were diagnosed in five patients with primary cSCC and in three patients with recurrent cSCC (Table 2). No distant metastasis was observed. Mean follow-up was 22 months (1–84).

**Table 1:** Primary and recurrent cSCCe in accordance to location

Location	Primary cSCC	Recurrent cSCC	All
Auricle	26 (90 %)	3 (10 %)	29 (100 %)
Lip	17 (77 %)	5 (23 %)	22 (100 %)
Nose	11 (73 %)	4 (27 %)	15 (100 %)
Buccae	8 (67 %)	4 (33 %)	12 (100 %)
Temple	8 (89 %)	1 (11 %)	9 (100 %)
Scalp	4 (67 %)	2 (33 %)	6 (100 %)
Ocular	2 (50 %)	2 (50 %)	4 (100 %)
Forehead	2 (100 %)	0	2 (100 %)
Neck	1 (100 %)	0	1 (100 %)
All	79 (79 %)	21 (21 %)	100 (100 %)

**Table 2:** Neck dissection—primary cSCC/recurrent cSCC

ND/pSCC/rSCC	Primary cSCC	Recurrent cSCC
Neck dissection	25	7
Meta	5	3

The most common tumour site was the auricle (29 cases; 29 %), followed by lip (22; 22 %), nose (15; 15 %), buccal region (12; 12 %), temple (9; 9 %), scalp (6; 6 %), ocular region (4; 4 %), frontal region (2; 2 %) and skin on the neck (1; 1 %) (Table 3). Neck dissection was most common in patients with lip tumours (17/22 patients; 77 %). Neck metastasis was found most commonly in patients with lip (2 patients) and buccal (2 patients) cSCC (Table 4).

**Table 3:** Location—stage differentiation

	Auricle	Lip	Nose	Buccae	Temple	Scalp	Ocular	Forehead	Neck	All
T1	6	8	2	3	2	1	2	0	0	24 (24 %)
T2	17	9	6	6	4	2	0	1	1	46 (46 %)
T3	5	4	5	1	3	3	1	1	0	23 (23 %)
T4	1	1	2	2	0	0	1	0	0	7 (7 %)
All	29	22	15	12	9	6	4	2	1	100 (100 %)

**Table 4:** Location—neck dissection and confirmed neck metastases

Location	Number	Neck dissection	Metastases
Auricle	29	5	1/29; 3 %
Lip	22	17	2/22; 9 %
Nose	15	3	1/15; 6 %
Buccae	12	4	2/12; 17 %
Temple	9	2	1/9; 11 %
Scalp	6	0	0
Ocular	4	1	1/4; 25 %
Forehead	2	0	0
Neck	1	0	0
All	100	32	8; 8 %

In males, the most common localisation was the auricle (25 of 66 cases; 38 %) and the lip (16 cases; 24 %). In females, the most common location was the nose (8 of 34 patients; 23 %) and buccal region (8 cases; 23 %) (Table 5). Neck dissection was performed in 20 male patients (30 %) and 12 female patients (35 %), with confirmed neck metastases in 5 females (15 %) and 3 males (5 %) (Table 6).

**Table 5:** Tumour location by patient sex

	Males	Females	All
Auricle	25 (87 %)	4 (13 %)	29 (100 %)
Lip	16 (62.5 %)	6 (37.5 %)	22 (100 %)
Nose	7 (47 %)	8 (53 %)	15 (100 %)
Buccae	4 (33 %)	8 (67 %)	12 (100 %)
Temple	7 (78 %)	2 (22 %)	9 (100 %)
Scalp	5 (83 %)	1 (17 %)	6 (100 %)
Ocular	1 (25 %)	3 (75 %)	4 (100 %)
Forehead	0	2 (100 %)	2 (100 %)
Neck	1 (100 %)	0	1 (100 %)
All	66 (66 %)	34 (34 %)	100 (100 %)

**Table 6:** Neck dissection by patient sex

ND/sex	Females	Males
Neck dissection	12/34 (35 %)	20/66 (30 %)
Metastases	5 (15 %)	3 (5 %)

The most common tumour stage was T2 (46 patients; 46 %) followed by T1 (24; 24 %), T3 (23; 23 %) and T4 (7; 7 %). Most of the patients who underwent neck dissection were those with T2 tumours (15 out of 46 patients; 32 %), followed by T3 (7/23; 30 %) and T4 (4/7; 57 %). Neck metastases were diagnosed in 4 out of the 46 patients (9 %) with stage T2 tumours, 3 out of 23 patients with stage T3 (13 %), and in 1 of the 7 patients with stage T4 (14 %) (Table 7).

**Table 7:** Neck dissection & metastases—TNM staging

	T1	T2	T3	T4	All
Neck dissection	5	15	7	4	32
Metastases	0	4	3	1	8

The most common histological tumour stage was G2 (57 cases; 57 %), followed by stage G3 (24; 24 %), and finally stage G1 (19 cases; 19 %). Of the 8 patients with confirmed neck metastasis, the tumour was poorly-differentiated in 4 of the 24 patients with stage G3 tumours (17 %), moderately-differentiated in 2 of the patients with stage G2 tumours (3 %), and highly-differentiated in the remaining stage G1 patients (2/19; 10 %) (Tables 8, 9).

**Table 8:** Location by cell differentiation

Location/grading	G1	G2	G3	All
Auricle	3	18	8	29
Lip	6	12	4	22
Nose	4	9	2	15
Buccae	2	6	4	12
Temple	3	5	1	9
Scalp	1	2	3	6
Ocular	0	2	2	4
Forehead	0	2	0	2
Neck	0	1	0	1
All	19 (19 %)	57 (57 %)	24 (24 %)	100

**Table 9:** Neck metastases in accordance to tumour differentiation

	G1	G2	G3	All
Metastases	2	2	4	8

Clinical examination of the neck was negative in all but one patient with T4 tumour. However, by using neck ultrasound (US) as a complementary examination, 32 patients were identified with suspected nodal metastasis. All 32 of these 32 patients underwent neck dissection, and neck metastasis was confirmed in 8 cases (25 %). Six patients were staged as N2b (TNM classification) and 2 as N2a (Table 10).

**Table 10:** Preoperative ultrasound with confirmed postoperative neck metastases

Case	USG
1. Case T2	Reactive lymph nodes
2. Case T2	Suspicion of metastatic lymph nodes
3. Case T2	Suspicion of metastatic lymph nodes
4. Case T2	Suspicion of metastatic lymph nodes
1. Case T3	Suspicion of metastatic lymph nodes
2. Case T3	Suspicion of metastatic lymph nodes
3. Case T3	Suspicion of metastatic lymph nodes
1. Case T4	Suspicion of metastatic lymph nodes

Of the eight patients with confirmed neck metastases, two had been previously treated for BCC on the face, and one patient had more than one cSCC tumour in the head and neck region. Taking into consideration other risk factors such as non-skin malignancies, immunosuppression, and tobacco, no influence on neck metastasis was observed (Table 11).

**Table 11:** Metastases with respect to other risk factors

History	All	Metastases
Other malignancy	8	0
Facial BCC	22	2
Multi SCC	16	1
Tobacco	19	0
Immunosuppression	2	0

Neck metastases were located as follows: in three patients: the parotid and neck level II (temple, auricle, and eye, respectively): in three other patients at levels I and II (lip in 2 cases, buccal region in the third case); in 1 case at level I (buccal area); and in patient at levels II, III and IV (the nose) (Table 12).

**Table 12:** Location of the neck metastases

Patient	Neck metastases
1	I, II
2	I, II
3	Parotid + II
4	Parotid + II
5	Ia + Ib
6	I, II
7	Parotid + II
8	II, III, IV

One patient died at second postoperative day due to polycythemia related complications. No other major complications were observed.

## Discussion

In our study, neck metastasis was diagnosed in 5 of the 79 patients with primary cSCC (6 %) and in 3 of the 21 patients with local recurrence (14 %). These findings agree with those reported by other authors. Del Regato et al. [6] reported that local recurrence increases the risk of neck metastasis, and Guruditt et al. [7] and Veness et al. [8] both reached a similar conclusion. In addition, Veness et al. [8] found that local recurrence of cSCC was a criteria for high risk of developing nodal metastasis. Other authors report that local recurrence accounts for 51 % of all neck metastases [9]. Similarly, Rowe et al. [1] found that local recurrence increased the risk of neck metastasis by 30 %.

In our study, the most common head and neck locations for cSCC were the auricle and lip. Baker et al. [10] reported that the auricle was the most common site for cSCC in their study of 227 patients. In a sample of 103 patients diagnosed with cSCC, Kosec et al. [11] also reported that the auricle was the most common location. In our study, the three most common locations for cSCC metastasis were the ocular, buccal, and lip regions, respectively. However, the number of patients with metastatic lesions was too small to form any definitive conclusions. Mourouzis et al. [12] reported that the auricle is the site that is associated with the highest risk of subsequent neck metastasis. In contrast, Vartanian et al. [13] found that lip tumours had the highest risk of neck metastasis, as did Rowe et al. [1], who reported a 14 % increased risk of neck metastasis in such patients. According to Vaness et al. [8] tumours located around the parotid gland (auricle, temple, frontal region) are associated with the highest risk of neck metastasis. They also report that scalp tumours have a worse prognosis due to the risk of transit metastases, although this finding has not yet been corroborated by any other authors.

In our study, nearly two-thirds of the patients were males and the most common tumour locations in that group were the auricle and lip, respectively. In contrast, in females, the most common localisations were the nose and buccal regions, each of which accounted for just under one-quarter of all tumour sites. Mean male age at diagnosis was 73.8 years, within females 76 years. Interestingly, neck metastasis was more common in the females (5 cases) than in the males, despite the small number of female patients. However, this observation is not significant given the small sample size of metastatic patients. Other authors have reported similar findings with regards to the predominance of cSCC tumours in males (Barzilai et al. [14]; Wang et al. [15]) and in the differences in primary tumour location by patient sex (Brougham et al. [16]). McLean et al. [17] assessed the influence of sex on neck metastasis and although they found, just as we did, that females had a higher percentage of neck metastases, they were unable to reach any definitive conclusions due to an insufficient sample size of metastatic female patients. However, this observation of a greater propensity for metastasis in females merits attention in future studies.

Ch’ng et al. [18] examined features of the primary tumour that could influence neck metastasis. Those authors evaluated tumour size, location, and differentiation and found that only poorly-differentiated tumours seemed to be associated with neck metastasis. Veness et al. [8], in their article on high risk cSCC, found that, by itself, tumour dimension is only a very weak prognostic factor for neck metastasis in comparison to cell differentiation. Similar results regarding the prognostic value of differentiation were reported by Breunninger et al. [19]. In contrast to these aforementioned findings, Rowe et al. [1] found that tumour size greater than 2 cm in diameter increased the risk of neck metastasis by 30 %. Our own data point to a possible relation between TNM classification and neck metastasis, with metastasis occurring in 9, 13, and 14 %, respectively, of patients with stage T2, T3, and T4 tumours. There were no neck metastases in patients with stage T1 tumours. Similar to the aforementioned authors, we found that poorly-differentiated tumours accounted for the majority of neck metastases (4 of the 8 patients with metastasis had poorly-differentiated tumours). Neck ultrasound (US) was a valuable complement to diagnosis of metastatic lesions in our sample. The role of ultrasound in clinical evaluation of the neck is well-known [20, 21] and when combined with radiographic examination, it can help to diagnose neck metastasis in 75 % of all cases [22, 23].

Three of the patients who had confirmed neck metastasis had other risk factors that may be relevant: 2 patients had received previous treatment for facial BCC and 1 patient had been diagnosed with more than one head and neck cSCC. No neck metastasis was diagnosed in patients with other risk factors (immunosuppression, tobacco use, non-skin malignancy) even though several authors report that those risk factors are associated with metastasis [1, 4, 8].

In our group of patients, tumours located in the temporo-ocular region metastasized mostly to the parotid gland and neck level II (3 out of 8 cases) whereas tumours around the lip and buccal region metastasized mainly to neck levels I and II (4 out of 8 patients). In one patient with an advanced tumour located on the nose, metastases were found in neck levels II, III and IV. Shah et al. [24] reported that tumours located frontally to the tragus of the auricle mostly metastasize to the parotid gland, pre-auricular lymph nodes, and to the anterior triangle lymph nodes of the neck. In contrast, tumours located posteriorly to the tragus mostly give rise to metastases to the post-auricular, suboccipital, and posterior triangle lymph nodes. Jol et al. [25] showed that tumours in the auricular, temporal and frontal region most frequently produce metastases to the parotid, while tumours in the buccal region result in metastases to neck levels I and II.

## Conclusion

Although cutaneous SCC metastasizes much less frequently to the neck lymph nodes than does mucosal SCC, precise evaluation of the neck should not be omitted in patients with cSCC. Despite the limitations of this study, mostly related to the low number of patients with confirmed neck metastases, our data—if considered together with the results published by other authors—seem to show that factors such as local recurrence, degree of cell differentiation, tumour dimension and/or location, can increase the risk of neck metastases. In patients with the presence of the aforementioned risk factors, neck dissection should be considered to evaluate for metastatic lesions. Neck ultrasound (US) is a valuable supplement to clinical examination and can aid in selecting patients for subsequent neck dissection.

## References

[1] Johnson TM, Rowe DE, Nelson BR, Swanson NA (1992). Squamous cell carcinoma of the skin (excluding lip and oral mucosa). J Am Acad Dermatol.

[2] Kyrgidis A, Tzellos TG, Kechagias N, Patrikidou A (2010). Cutaneous squamous cell carcinoma (SCC) of the head and neck: risk factors of overall and recurrence-free survival. Eur J Cancer.

[3] Karia PS, Han J, Schmults CD (2013). Cutaneous squamous cell carcinoma: estimated incidence of disease, nodal metastasis, and deaths from disease in the United States, 2012. J Am Acad Dermatol.

[4] Martinez JC, Cook JL (2007). High-risk cutaneous squamous cell carcinoma without palpable lymphadenopathy: is there a therapeutic role for elective neck dissection?. Dermatol Surg.

[5] Edge SB, Compton CC (2010). The American joint committee on cancer: the 7th edition of the AJCC cancer staging manual and the future of TNM. Ann Surg Oncol.

[6] Del Regato JA, Vuksanovic M (1962). Radiotherapy of carcinomas of the skin overlying the cartilages of the nose and ear. Radiology.

[7] Gurudutt VV, Genden EM (2011) Cutaneous squamous cell carcinoma of the head and neck. J Skin Cancer. doi:10.1155/2011/502723 10.1155/2011/502723 PMC3064996 21461387

[8] Veness MJ (2007). High-risk cutaneous squamous cell carcinoma of the head and neck. J Biomed Biotechnol.

[9] Tavin E, Persky M (1996). Metastatic cutaneous squamous cell carcinoma of the head and neck region. Laryngoscope.

[10] Baker NJ, Webb AA, Macpherson D (2001). Surgical management of cutaneous squamous cell carcinoma of the head and neck. Br J Oral Maxillofac Surg.

[11] Kosec A, Svetlina L, Luksic I (2013). Significance of clinical stage, extent of surgery and outcome in cutaneous squamous cell carcinoma of the head and neck. Int J Oral Maxillofac Surg.

[12] Mourouzis C, Boynton A, Grant J, Umar T (2009). Cutaneous head and neck SCCs and risk of nodal metastasis e UK experience. J Craniomaxillofac Surg.

[13] Vartanian JG, Carvalho AL, de Araujo Filho MJ, Junior MH (2004). Predictive factors and distribution of lymph node metastasis in lip cancer patients and their implications on the treatment of the neck. Oral Oncol..

[14] Barzilai G, Greenberg E, Cohen-Kerem R, Doweck I (2005). Pattern of regional metastases from cutaneous squamous cell carcinoma of the head and neck. Otolaryngol Head Neck Surg.

[15] Wang JT, Palme CE, Morgan GJ, Gebski V (2012). Predictors of outcome in patients with metastatic cutaneous head and neck squamous cell carcinoma involving cervical lymph nodes: improved survival with the addition of adjuvant radiotherapy. Head Neck.

[16] Brougham ND, Dennett ER, Cameron R, Tan ST (2012). The incidence of metastasis from cutaneous squamous cell carcinoma and the impact of its risk factors. J Surg Oncol.

[17] McLean T, Brunner M, Ebrahimi A, Gao K (2013). Concurrent primary and metastatic cutaneous head and neck squamous cell carcinoma: analysis of prognostic factors. Head Neck.

[18] Ch’ng S, Clark JR, Brunner M, Palme CE (2013). Relevance of the primary lesion in the prognosis of metastatic cutaneous squamous cell carcinoma. Head Neck.

[19] Breuninger H, Black B, Rassner G (1990). Microstaging of squamous cell carcinomas. Am J Clin Pathol.

[20] Hodder SC, Evans RM, Patton DW, Silvester KC (2000). Ultrasound and fine needle aspiration cytology in the staging of neck lymph nodes in oral squamous cell carcinoma. Br J Oral Maxillofac Surg.

[21] Nemec SF, Linecker A, Czerny C, Imhof H (2008). Detection of cutaneous invasion by malignant head and neck tumors with MDCT. Eur J Radiol.

[22] van den Brekel M, Castelijns J, Stel H (1993). Modern imaging techniques and ultrasound-guided aspiration cytology for the assessment of neck node metastases: a prospective comparative study. Eur Arch Otorhinolaryngol.

[23] Yucel T, Saatci I, Sennaroglu L (1997). MR imaging in squamous cell carcinoma of the head and neck with no palpable lymph nodes. Acta Radiol.

[24] Shah JP, Patel SG, Singh B (2012) Jatin shah’s head and neck surgery and oncology, Chap 3, 4th Edn. Elsevier, Philadelphia

[25] Jol JA, van Velthuysen ML, Hilgers FJ, Keus RB (2003). Treatment results of regional metastasis from cutaneous head and neck squamous cell carcinoma. Eur J Surg Oncol.

